# Postsurgical Pathologies Associated with Intradural Electrical Stimulation in the Central Nervous System: Design Implications for a New Clinical Device

**DOI:** 10.1155/2014/989175

**Published:** 2014-04-01

**Authors:** Katherine N. Gibson-Corley, Oliver Flouty, Hiroyuki Oya, George T. Gillies, Matthew A. Howard

**Affiliations:** ^1^Division of Comparative Pathology, Department of Pathology, Carver College of Medicine, University of Iowa, 500 Newton Road, 1167 ML, Iowa City, IA 52242, USA; ^2^Department of Neurosurgery, Carver College of Medicine, University of Iowa, 500 Newton Road, 1167 ML, Iowa City, IA 52242, USA; ^3^Department of Mechanical and Aerospace Engineering, University of Virginia, Charlottesville, VA 22904, USA

## Abstract

Spinal cord stimulation has been utilized for decades in the treatment of numerous conditions such as failed back surgery and phantom limb syndromes, arachnoiditis, cancer pain, and others. The placement of the stimulating electrode array was originally subdural but, to minimize surgical complexity and reduce the risk of certain postsurgical complications, it became exclusively epidural eventually. Here we review the relevant clinical and experimental pathologic findings, including spinal cord compression, infection, hematoma formation, cerebrospinal fluid leakage, chronic fibrosis, and stimulation-induced neurotoxicity, associated with the early approaches to subdural electrical stimulation of the central nervous system, and the spinal cord in particular. These findings may help optimize the safety and efficacy of a new approach to subdural spinal cord stimulation now under development.

## 1. Introduction

Over the past few years we have been developing a method of spinal cord stimulation (SCS) that is designed to enable more precise activation of targeted pathways within the human spinal cord [1, 2]. Central to this approach is the Human Spinal Cord Modulation System (HSCMS), also referred to as the “I-Patch” device. It is a subdural implant the platinum electrodes of which are positioned directly on the pial surface of the spinal cord. This configuration offers the advantage of minimal shunting of the electrical stimulation currents by the relatively high conductivity cerebrospinal fluid (CSF) which has long been known to limit the performance of standard epidural SCS devices [3]. By widening the therapeutic window for stimulation, the HSMCS also offers the potential advantage of more selective activation of the spinal cord fibers while avoiding excitation of nearby nontargeted structures, for example, the dorsal rootlets [4]. Of course, much is still unknown about the specific functions of several of the fiber tracts within the spinal cord. However, successful achievement of these goals would make it possible to begin testing for improved therapeutic efficacy via more selective targeting of SCS, especially for the treatment of intractable pain, a problem that at present is inadequately addressed by epidural devices in up to half of all cases [5]. Future versions of the HSCMS will also incorporate penetrating electrodes into the device design thus allowing access to motor control pathways positioned deeply within the spinal cord parenchyma.

Because it is subdural, the HSCMS offers a number of distinct potential advantages over the existing devices used for SCS that are either percutaneously placed or implanted via an open surgical procedure. For instance, by keeping the HSCMS electrodes fixed in place on the dorsal pial surface, there is no need for a control algorithm that constantly adjusts the stimulus intensity as the spinal cord moves within the thecal sac. Moreover, while epidural devices are only able to stimulate fibers within a thin layer (~250 *μ*m thick) beneath the pial surface [6], modeling studies have now shown that subdural stimulation will permit a much deeper and well-focused penetration by the electrical stimulation fields [7]. This latter point opens the door to the interesting scientific possibility of using the HSCMS to obtain fundamental neurophysiological information about the somatotopic organization of the axonal fiber tracts within the spinal cord. Of course, any such benefits will come at the expense of a more complex neurosurgical procedure (durotomy, to expose the spinal cord), the attendant need for a robust postimplant seal of the durotomy incision (to prevent CSF leaks), and design requirements that will allow the HSCMS to function safely within the spinal canal on a permanent basis.

Because the HSCMS revisits the original clinical approach to spinal cord stimulation, in which the electrodes or electrode arrays were implanted subdurally, it is important to understand the reasons why that approach eventually gave way in the 1970s to the present paradigm of epidural implantation. Principal among those reasons was the less invasive nature offered by epidural implantation, while still maintaining ability to generate therapeutic effects within the dorsal columns. Additional issues involved certain surgical difficulties and pathologies that were encountered by several surgical groups that carried out placement of subdural spinal cord stimulators through about 1975. While most such surgeries were successful, the types of difficulties that arose from time to time included spinal cord injury and/or vascular compromise, infection, cerebrospinal fluid leakage, hematoma formation, and chronic formation of fibrosis or scar tissue. These are described in the contemporary literature of that period covering both animal models of subdural spinal cord stimulation and its rather widespread use in patients, which we review here. We then go on to discuss how these factors were considered when designing a HSCMS that is intended to safely and effectively deliver electrical stimuli directly to the human spinal cord.

## 2. Background: Previous Clinical Use of Subdural Devices for SCS

**Figure 1:**
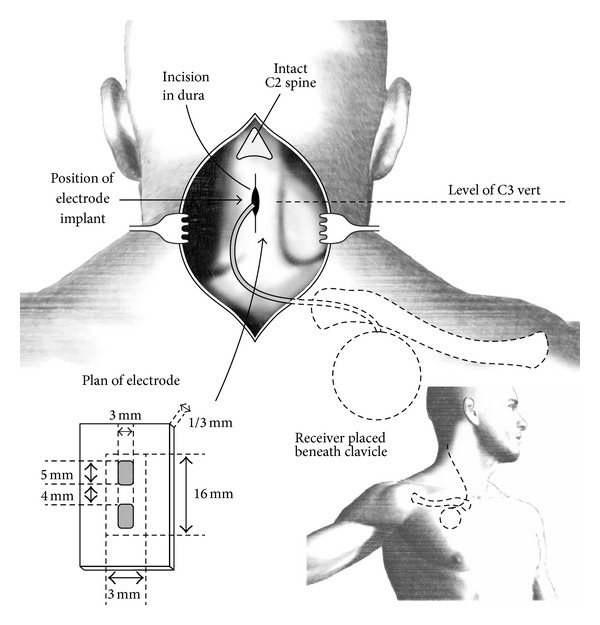
An example of one of the original surgical approaches for placement of an intradural spinal cord stimulator array, after Figure 5 of [17] and after Figure 24.3 of [15]. (Reprinted with permission of J. G. Wepsic, M.D., Thieme Medical Publishers, Inc., and The Congress of Neurological Surgeons.)

The original implementations for SCS therapies typically employed subdural devices, even with substantial evolution of the technique over the first decade of use. Referred to as “dorsal column stimulation” at its inception, Shealy and colleagues [8–10] and other early adopters [11–16] used simple electrode configurations often consisting of either monopolar or bipolar platinum contacts mounted on silicone-dacron bases that were inserted in place inside the dura.  Figure 1, from [15, 17], shows an example of the typical surgical arrangement employed at the time. The details of another contemporary operative procedure (subdural extra-arachnoid placement) are described by Nashold and Friedman [12]. In addition to the early clinical trials carried out in the USA, a large number of subdural dorsal column stimulators were implanted in patients in Canada [18], England [19, 20], France [21, 22], Germany [23–25], and Sweden [26].

These implantations were carried out to treat patients with numerous chronic pain conditions including those arising from failed back surgery, phantom limb syndromes, arachnoiditis, cancer, and other etiologies. The typical risks associated with subdural placement that were discussed in the research articles and case reports of the day included occasional CSF leaks at the point where the leads exited the dura resulting in CSF fistula formation, spinal cord compression, and, rarely, infections such as meningitis (see, e.g., [11]). From a technical hardware standpoint, the difficulties that were encountered included the potential for lead breaks, failures in the subcutaneous pulse generators, and upward drift in the required stimulus voltage. The latter issue was presumably associated with slowly developing increases in the electrode impedances, possibly due to chronic collagen encapsulation [27]. In spite of these difficulties, it was recognized that subdural placement of the electrodes improved electrical coupling to the spinal cord [28] and was particularly important when the specific site of stimulation on the dorsal columns was critical [29]. Also, Shealy [30] pointed out that none of the stimulator placements, including their subdural approach, had resulted in any known electrical stimulation-induced damage to the spinal cord. (It is interesting to note that Hosobuchi et al. even deployed intraparenchymal penetrating electrodes into the spinal cord for stimulations during screening procedures [31].)

**Figure 2:**
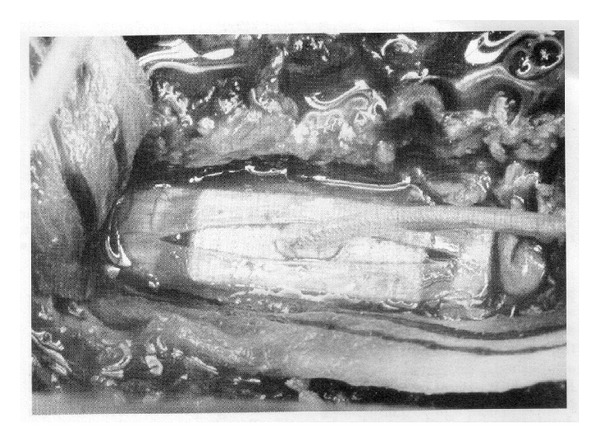
An example of the endodural approach to placement of a spinal cord stimulator array in a patient, after Figure 3 in [29]. (Reprinted with permission of C. Burton, M.D., and Elsevier Inc.)

Even so, the need to open the dura and the complications that sometimes arose led to an eventual shift in the surgical methods used, away from subdural and towards epidural placement of the electrodes. During the transition period, some surgeons placed electrodes within endodural pouches created by separating the layers of the dura [32], as shown in Figure 2 [29]. By the 1980s, the epidural placement strategy had become established as the dominant practice, and this anatomical space is where all stimulator leads are placed at present. Some details of the instrumentation (electrodes, pulse generators) used at the time of the subdural and endodural placements are given by Burton [28] and Shealy [30]. Burton [28] and Taub [33] note that during that era, that is, through about 1974, some 3,000 patients had been treated with dorsal column stimulators. Roughly 25% of those cases are reported in the literature from that period and the remaining 75% of the cases likely followed a similar breakdown of methods, but the lack of a comprehensive, published database makes it impossible to discern. The clinical results during this early stage, in terms of efficacy of pain relief, were mixed. Some of the larger studies reporting results were those of Shealy [30], Long and Hagfors [34], Burton [27], and Erickson [35].

As mentioned above, these early subdural implantation studies now set the stage for the reintroduction of the technique. The lessons learned then from subdural placement of the electrodes (excellent electrical coupling to the spinal cord, improved site-specific stimulations), in conjunction with the availability of modern neurosurgical materials and technology, suggest that this approach should be reinvestigated. In particular, the ongoing clinical success of the closest predicate device, the auditory brainstem implant, speaks directly to the potential for the safe and efficacious long-term implantation of a stimulation device directly on the pial surface of the central nervous system's tissues [36, 37]. The HSCMS has been designed to take advantage of these contemporary factors and address the shortcomings of the existing epidural approaches to SCS, now employed in about 35,000 patients per year [38]. In developing either the HSCMS or any other new approach to intradural SCS, it will be important to understand how the results of pathological studies of spinal cord tissue can be used to aid in the design of a maximally effective and safe device. Therefore, we now review the relevant clinical and experimental laboratory findings within that context.

## 3. Key Pathologic Findings Related to Subdural Stimulation

**Figure 3:**
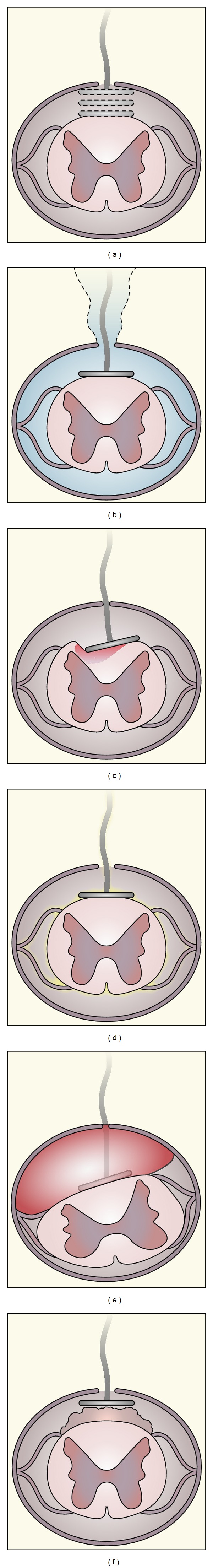
Summary of the types of neurosurgical complications reported during the early clinical use (ca. 1970) of intradural spinal cord stimulators in patients. (a) The baseline situation is shown in which the intradural array has been implanted either immediately under the dura, within the CSF layer, or directly on the dorsal surface of the spinal cord, with its leads traversing the dura, which forms a seal around them. (b) The difficulty encountered most frequently is shown: leakage of the CSF at the point where the leads traverse the dura. (c) Spinal cord contusion. (d) Infection within the spinal canal. (e) Formation of an intradural hematoma compressing the spinal cord. (f) Chronic formation of scar tissue or mature fibrous tissue around the stimulator.

The principal kinds of surgical challenges and postsurgical pathologies encountered for subdural stimulator implantation are summarized in Figure 3. The general arrangement for subdural placement is shown in Figure 3(a) which, by inference, is meant to depict the range of electrode positioning methods (subdural through endodural) that were practiced at the time.

### 3.1. Cerebrospinal Fluid Leakage

Some of the early workers reported acute pathology arising from cerebrospinal fluid (CSF) leakage due to incomplete healing of the durotomy at the implantation site, presenting as subcutaneous swelling around the electrode cable at the traversal point through the dura (Figure 3(b)) [12, 32, 39–41]. In general, CSF leakage is often effectively managed through either a follow-up surgical reapproximation of the dura or by using a temporary lumbar drain CSF diversion method [42].

### 3.2. Vascular Disruption and/or Spinal Cord Contusion

Acute pathology associated with the* in situ* physical presence of intradural SCS devices would include vascular congestion and stasis. For instance, transient spinal cord compression secondary to electrode placement (Figure 3(c)) was observed in one patient case where a subarachnoid electrode was implanted [43].

**Figure 4:**
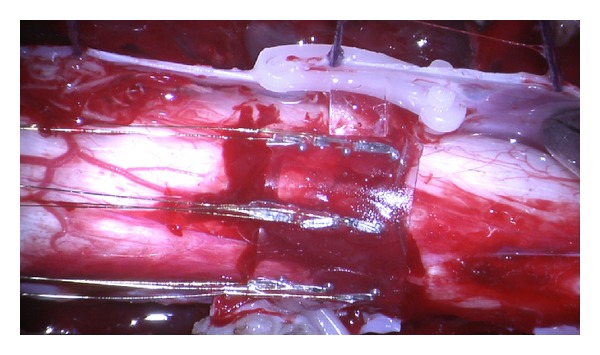
The electrode-bearing surface of an early prototype version of the HSCMS. The device is shown in place on an exposed section of ovine spinal cord during an acute* in vivo* trial. This particular device is made of a nearly transparent thin film of silicone, had nine electrodes arranged in a 3 × 3 array, and was held in place by clips attached to dural flaps.

In general, if an implant is to be placed directly upon the pial surface of the spinal cord, it should conform concisely to the cord's morphology so as to not disrupt blood flow through the surface and immediate subsurface vessels. A recent study in sheep using an early prototype of the HSCMS/I-Patch spinal cord stimulator revealed that there were no vascular lesions or evidence of vascular congestion in vessels along the dorsal portion of the pia or cord directly in contact with the device [4]. In that work, as seen in Figure 4, the electrode-bearing membrane of the prototype was draped gently over the spinal cord surface and its bilateral attachment arms were secured to the dentate ligaments in a way that did not directly compress the underlying neural tissues [44].

### 3.3. Infection

Localized infection (Figure 3(d)), sepsis, meningitis, arachnoiditis at the site of the electrode, extradural hematomas, CSF fistulas, pseudomeningocele formation, and spinal cord injury have all been documented with subdural spinal cord stimulation [32, 39, 40, 45]. There are also reports of extrusion of the lead wires through the skin which subsequently led to infection [39, 40]. Although most infections associated with implantation of central nervous system electrical stimulation devices occur soon after surgery and can be addressed by removing the device, a localized infection has been documented to occur years after implantation in a sacral anterior root stimulator case [46]. The inciting causes of these infections are rarely reported, although in one case* Staphylococcus aureus* was determined to be the pathogen which appeared to be present within the instrument itself, presumably from a contaminated implant [39].

### 3.4. Hematoma Formation

**Figure 5:**
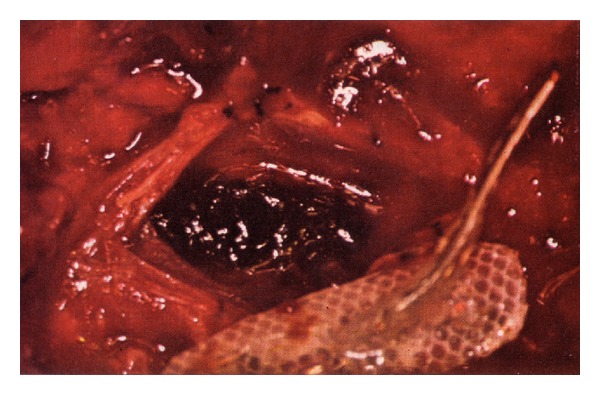
Hematoma produced by possible laceration of a pial vessel underneath the electrode bearing surface of a subdural spinal cord stimulator, after the figure in [47]. (Reprinted with permission of P. J. Grillo, M.D. and The American Medical Association.)

Hematoma formation secondary to subdural stimulator placement can occur (Figure 3(e)). There is a single report of a patient with an intradural dorsal column stimulator which, 18 months after surgery, developed a hematoma directly under the electrodes. This hematoma, shown in Figure 5, compressed the underlying cord and led to acute clinical signs. Interestingly, this stimulator had not been in use for about a year, thus excluding delivery of electrical stimuli as the possible cause of hematoma formation [47].

### 3.5. Chronic Fibrosis

Chronic pathology occurs over several days to months, and even years. Therefore, it is often difficult to determine if the surgical procedure or the presence of the electrodes (mechanical pathology) for a long period of time was the inciting cause of chronic injury. A very common finding in long-term animal studies employing chronic electrical stimulation and the presence of subdural electrodes is fibrosis and meningeal thickening (Figure 3(f)). This finding is noted both when using extradural stimulators [48] and those placed subdurally [29, 49]. The mere presence of the electrodes appears to lead to meningeal thickening [49], although the extent of fibrosis is also affected by electrical stimulation, at least in the case of extradural stimulation [48]. An animal study by Yuen et al. [50] concluded that the mere presence of inactive electrodes led to meningeal fibrosis and minimal cortical compression. Burton [29] investigated this point by implanting dural patches of Teflon (used commonly at the time as the mounting surface for intradural electrodes) on the cerebral cortex of cats for periods of 55 to 90 days. He found that the implants were encased in thickened dura with a minimal inflammatory cell response. However, other investigators [51] found meningeal thickening or fibrosis only rarely in feline models with subdural electrodes implanted chronically for similar periods (8 to 16 weeks). The difference between these findings may be due to the materials that were used: in the latter study, the implants were of Parylene and other types of insulators. Meningeal thickening due to increased fibrotic tissue was also reported frequently secondary to electrical stimulation [49, 50, 52]. In one study, this fibrotic response was markedly increased in sites that received electrical stimulation as opposed to negative control sites. The extent of this fibrosis was proportional to the electric charge delivered, indicating that subdural electrical stimulation can specifically incite meningeal fibrosis [49].

In patients, the best documented chronic complication of subdural electrode placement is fibrosis or thickening of the arachnoid surrounding the implanted electrodes [12, 39, 40]. The opportunity to study these pathological changes is afforded by a clinical scenario that necessitates surgical removal of a device. Marked thickening of the arachnoid can lead to formation of an insulating barrier between the stimulating electrode and the spinal cord, resulting in lead insulation and poor transmission of current to the spinal cord targets, and concomitant deterioration in clinical efficacy [39, 40]. Formation of excessive fibrous tissue has also been well described in other neural stimulation devices, including cochlear implants located in the middle ear [53]. Interestingly, in the case of cochlear implants, this fibrotic tissue can become ossified and lead to significant temporal bone thickening [53, 54]. However, to our knowledge, ossification has not been described in the case of implanted intradural spinal cord stimulators.

### 3.6. Pathology Induced by Electrical Stimulation

Electrical stimulation-induced neurotoxicity is a distinct pathologic entity to consider when evaluating the effects of a spinal cord stimulation system. There is a vast amount of literature on stimulation-induced damage to CNS tissues in general, with McCreery et al. [55] and Shannon [56] having made early quantitative assessments of the nature of tissue damage in terms of the charge density and the charge per phase during a stimulation cycle.

The device parameters that govern the stimulation process include the voltage across electrodes, the resulting current flow within the tissues, the frequency, duration, and duty cycle of the pulses, and the contact area, composition, and configuration (monopolar, bipolar, etc.) of the electrodes. Charge-balanced pulses are typically employed during* in vivo* studies in order to maintain net electrical neutrality within the tissues, and the intercontact impedance provides an important window into the electrophysiological response of the tissues to the applied stimuli. Platinum electrodes are used most commonly, as it has been shown that they are biocompatible, do not elicit a foreign body response, and corrode only over decades thus making their surfaces stable in a fluid environment [57]. Shannon's work [56], for example, coupled all of these factors together to yield an expression for the damage threshold current:

(1) I=(d2T)·(π·10k)1/2,

where *I* is the current,* d* is the diameter of the (disc-shaped) electrode,* T* is the duration of a single phase of the stimulation cycle, and *k* is the slope of the log-log plot of the charge density versus charge per phase curve for a given stimulation system and type of tissue. Numerical values of *k* ≳ 1.5 are empirically associated with tissue damage.

There is a vast literature on electrical stimulation-induced neuropathology, and it is beyond the scope of our present work to review it here. We note only that the key histopathologic findings secondary to electrical stimulation of the central nervous system include increased permeability of blood vessels [58], vascular congestion and thrombosis [57, 58], neuronal process swelling and necrosis [57–59], localized gliosis [50], and myelin degeneration [49, 52]. Interestingly, there are also reports in which no significant histopathological findings were found following electrical stimulation [60, 61].

## 4. Discussion

### 4.1. General Findings

A primary reason for our assessment of the published pathologic findings associated with subdural electrical stimulation of the central nervous system was to make use of this information when designing a new kind of direct spinal cord stimulator. The evidence from human and experimental animal studies demonstrates that reactive changes occur when an implant is positioned on or within central nervous system tissue, irrespective of whether electrical stimuli are delivered through the device. With regard to the subdural spinal cord stimulators used in the early years of dorsal column stimulation for the treatment of intractable pain, the nature of these outcomes is summarized in Figure 3. Even when the implants are constructed of biocompatible materials, and the insertion technique is designed and implemented properly, it is clear that postimplantation pathological changes can sometimes be expected. However, in the absence of surgical complications, such as direct mechanical injury to the spinal cord during the insertion procedure, these changes are not associated with adverse clinical effects.

The pathological effects of electrical simulation on central nervous system tissue have been extensively investigated by researchers from multiple laboratories over many decades. Much of this work was motivated by the practical need to determine safe electrical stimulation parameters when designing human neural prosthetic devices. The resulting literature, encompassing work carried out using a range of complimentary investigative methods, provides clear evidence that electrical stimuli can be safely delivered directly to the spinal cord. This is achieved by using stimulus delivery parameters that are consistently identified across studies as being below tissue injury thresholds (e.g., a slope of *k* < 1.5 for a log-log plot of the charge density versus charge per phase values of the stimulation mode employed). Our own experimental studies, initially on the sensitive cortical surface of the brain, have also found no pathologies during the acute phase of electrical stimulation in an ovine model [4]. We are nevertheless presently extending that work to include a complete gross, histopathologic, and ultrastructural analysis of the spinal cord itself in animals implanted chronically with the HSCMS. The scope of that effort includes assessment of meningeal fibrotic response and vascular integrity of the spinal cord as a function of the stimulation parameters.

It would ideally be interesting to compare the categories and rates of the subdural implantation pathologies with those associated with the present epidural spinal cord stimulator implants. While there will inevitably be some overlap in types of categories (e.g., postoperative infections), making such a comparison in general is complicated in part by the limited descriptions of overall complication rates given in the early literature. Even so, it would be useful to know how the fundamental differences in the nature of the procedures (subdural versus extradural) and the significant differences between the early and modern technological designs of the devices fare even approximately in relation to each other in terms of pathological outcomes. However, given that only some 3000 subdural implantations were carried out through 1975 and that there have been some hundreds of thousands of epidural procedures since then [62], much further assessment of the literature and statistical analysis of the clinical findings will be needed to arrive at meaningful results. That effort is now underway and we will report the findings in future work.

### 4.2. Implications for the HSCMS Design

**Figure 6:**
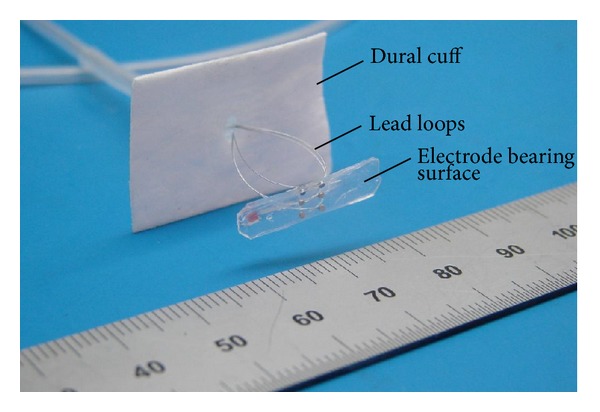
Photograph of the mechanical components of a prototype of the HSCMS which will be placed in the intradural space. The electrode-bearing surface will rest directly on the dorsal aspect of the spinal cord.

The development of a safe and effective direct spinal cord stimulation system using novel contemporary materials and design concepts is presently underway in our laboratories, and the significant advantages it offers over the existing epidural approaches are discussed in detail elsewhere [2, 4]. The original subdural SCS implantations that were carried out through roughly 1975 yielded to epidural implants in part because the relative simplicity of the latter procedure made it attractive enough to offset the risk of reduced therapeutic efficacy arising from CSF shunting effects, drifts in lead position, and so forth. The technical issues sometimes associated with the original subdural implantation procedures, for example, CSF leakage due to incomplete seal of the dura around the implant's leads, have since been overcome by improvements in neurosurgical methods and materials. For instance, FDA-approved resorbable dural substitutes (e.g., Durepair or Dura-Guard, among others) have been developed and put into routine use, significantly improving the ability to obtain permanent water-tight seals of dural incisions.  Figure 6 is a photograph of a prototype HSCMS, of the kind presently being tested in our long-term ovine model. We are using prototypes such as these to demonstrate how the present state-of-the-art neurosurgical materials can be incorporated into a subdural SCS device, and evaluated for eventual implantation into human patients. The electrode bearing surface of this device rests directly on the dorsal aspect of the spinal cord. The loop-shaped leads from the six individual electrodes meet at a point on the underside the resorbable dural cuff and are bonded into a pass-through aperture in it. Following surgical implantation inside the durotomy, the dura is reapproximated to the dural cuff and sutured securely to it, with subsequent reformation of an integral layer as the dura and the dural cuff material fuse via an* in situ* scarring process.

Additional design features of the HSCMS enable it to either circumvent or overcome some of the other issues that had been noted with use of the original subdural devices, as well. For instance, Oliynyk et al. [63] demonstrated that with proper sizing of the diameter of the lead-loop wires, the pressure on the spinal cord exerted by the electrode bearing surface of the device due to the compliance of the lead loops can easily be kept within the range of normal intrathecal pressure (10 to 15 mm Hg), thus significantly reducing the risk of any transient spinal cord compression. Likewise, risk of fatigue and possible breakage of the lead wires is ameliorated substantially by the strain relief intrinsic to the lead loops. When compressed to their working size inside the thecal sac, they can accommodate the HSCMS/spinal cord axial movement during flexion without tautness or tethering [64] (Grillo et al. [47] had expressed concern about the restrictive or tethering effects of the earlier devices on spinal cord.) Lastly, the dorsal arc length subtended by the electrode bearing surface of the device is chosen to prevent it from making contact with the dorsal nerve rootlets, and its structural compliance and radius of curvature allow it to conform optimally to the spinal cord surface [65].

At this time, the closest modern predicate device to the HSCMS that is in routine clinical use is the auditory brainstem implant (ABI), which is a neuroprosthesis that is technologically related to the cochlear implant (CI). There have been many well designed studies of these devices reported in both animal models [66–70] and patients [71–75]. As briefly mentioned here, one of the primary pathologies that occured with ABI is CSF leak [76, 77], while with the CI it is temporal bone fibrosis and ossification [54]. Regarding the latter, although meningeal fibrosis is commonly seen at the site of implanted intradural electrodes in the central nervous system, there do not appear to be any reports of ossification of this tissue. Similar findings of fibrosis are also reported in animal studies with CIs [66, 70] and these reports also mention that there are rarely stimulus-induced changes to cochlear nucleus structures [69]. Overall, the implantation and use of ABI, which is the most relevant predicate device in our case, is seen to cause only minimal if any histopathologic damage, although this can be difficult to evaluate in patients with previous pathology related to hearing loss due to tumors of the acoustic nerve [72, 78].

## 5. Conclusions

One of our goals has been to review the published literature describing the acute and chronic histological changes that occur following placement and use of subdural central nervous system electrical stimulation devices. Many of the histological changes that were observed developed in reaction to placement of a mechanical implant on the surface, or within the substance of the brain or spinal cord, and are not associated with adverse clinical consequences. Similar histological changes also occur following implantation of other, nonstimulating devices into brain or spinal cord (e.g., silicone catheters for CSF diversion or the convection-enhanced delivery of agents). Over a period of decades, researchers have meticulously investigated the pathological changes specifically associated with the delivery of electrical stimuli into central nervous system tissue. The results of those studies provide consistent evidence of the safety of electrical stimulation below well-described tissue injury thresholds. This valuable information is being used to inform the design of a new type of direct spinal cord stimulation device that promises to be safe and effective inside the spinal canal.
